# Visual prosthesis wireless energy transfer system optimal modeling

**DOI:** 10.1186/1475-925X-13-3

**Published:** 2014-01-16

**Authors:** Xueping Li, Yuan Yang, Yong Gao

**Affiliations:** 1 Department of Electronic Engineering, Xi’an University of Technology, Jinhua Road No.5, Xi’an 710048, China

**Keywords:** Biological capacitance, Optimal modeling, Visual prosthesis, Wireless energy transfer, Planar spiral coils

## Abstract

**Background:**

Wireless energy transfer system is an effective way to solve the visual prosthesis energy supply problems, theoretical modeling of the system is the prerequisite to do optimal energy transfer system design.

**Methods:**

On the basis of the ideal model of the wireless energy transfer system, according to visual prosthesis application condition, the system modeling is optimized. During the optimal modeling, taking planar spiral coils as the coupling devices between energy transmitter and receiver, the effect of the parasitic capacitance of the transfer coil is considered, and especially the concept of biological capacitance is proposed to consider the influence of biological tissue on the energy transfer efficiency, resulting in the optimal modeling’s more accuracy for the actual application.

**Results:**

The simulation data of the optimal model in this paper is compared with that of the previous ideal model, the results show that under high frequency condition, the parasitic capacitance of inductance and biological capacitance considered in the optimal model could have great impact on the wireless energy transfer system. The further comparison with the experimental data verifies the validity and accuracy of the optimal model proposed in this paper.

**Conclusions:**

The optimal model proposed in this paper has a higher theoretical guiding significance for the wireless energy transfer system’s further research, and provide a more precise model reference for solving the power supply problem in visual prosthesis clinical application.

## Background

Visual prosthesis is one of the hot research issues in biomedical field and how to solve the energy supply problem of implantable visual prosthesis is particularly important. To reduce the patient’s pain of multiple surgical implantable battery replacement, in recent years wireless energy transfer become scholar’s accepted method [1-9]. Many scholars have done some research on wireless energy transfer of the coupling coils, but the models built in these papers are too ideal, for example the internal resistance of power source and the parasitic capacitance of the coil are neglected, and most of them are based on air medium [1-3]. Part of the literatures have considered the biological tissue influence on energy transfer, but they all are based on the analysis of the simulation software such as Ansoft-HFSS without establishing more accurate mathematical model on the basis of the physical properties of biological tissue [4-8]. In this paper, we focus on the optimal modeling of visual prosthesis wireless energy transfer system, During the modeling, on one hand, the factors that may affect the wireless energy transfer are fully considered without taking many ideal assumptions. On the other hand, the influence of biological tissue, the coupling medium between primary coil and secondary coil, is fully taken account.

## Methods

### The ideal modeling of wireless power transfer system

**Figure 1:**
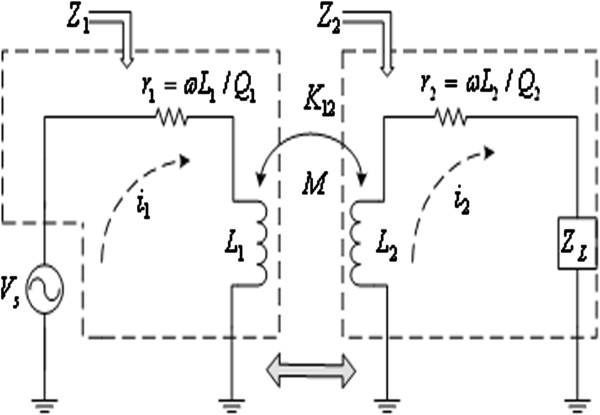
**The model of ideal wireless energy transfer system.** Figure 1 shows the model of ideal wireless energy transfer (wireless power transfer, WPT) system.

Figure 1 shows the model of ideal wireless energy transfer (wireless power transfer, WPT) system [1], in which the internal resistance of power source *r*_*s*_ and the coil parasitic capacitance are ignored. Energy transfer is achieved by the coupling of the primary inductance coil *L*_1_ and the secondary inductance coil *L*_2_. Usually the mutual inductance (factor) *M* or the coupling coefficient *K*_12_ characterizes the coupling degree of two coils, and the formula is $K_{12}=\frac{M}{\sqrt{L_{1}L_{2}}}$. In Figure 1, *V*_*s*_ means the energy source. *Z*_1_ means the equivalent impedance of the primary coil circuit, which is composed by the reactance *jωL*_1_ of the primary coil *L*_1_ (where *ω* is the energy transfer angular frequency) and equivalent series resistance *r*_1_, that is *Z*_1_ = *r*_1_ + *jωL*_1_. *Z*_2_ means the equivalent impedance of the secondary coil circuit, which is composed by the reactance *jωL*_2_ of the secondary coil *L*_2_, the equivalent series resistance *r*_2_ and the load *Z*_*L*_ (*Z*_*L*_ = *R*_*L*_ + *jωX*_*L*_), that is *Z*_2_ = *r*_2_ + *jωL*_2_ + *Z*_*L*_.

According to the energy conservation law, the total energy supplied by the energy source *V*_*s*_ is consumed by *r*_1_, *r*_2_ and *R*_*L*_ jointly, and the reactance parts of *L*_1_, *L*_2_ and *X*_*L*_ do not involved in energy consumption. Therefore, the energy transfer efficiency *η* of the wireless energy transfer system can be defined as the ratio of the energy consumed by the load and the total energy provided by the energy source. That is:

(1) $$\eta =\frac{P_{R_{L}}}{P_{R_{L}}+P_{r_{2}}+P_{r_{1}}}$$

In Formula (1),

$P_{R_{L}}=i_{2}^{2}R_{L}$, which is the energy obtained (consumed) by the load, *i*_2_ is the secondary coil loop current. $P_{r_{2}}=i_{2}^{2}r_{2}$, which is the energy consumption of the equivalent series resistance *r*_2_ in the secondary coil. $P_{r_{1}}=i_{1}^{2}r_{1}$, which is the energy consumption of the equivalent series resistance *r*_1_ in the primary coil, *i*_1_ is the primary coil loop current.

(2) $$i_{1}=\frac{Z_{2}V_{s}}{Z_{1}Z_{2}+{\left(\omega M\right)}^{2}}$$

(3) $$i_{2}=\frac{-j\omega MV_{s}}{Z_{1}Z_{2}+{\left(\omega M\right)}^{2}}$$

So, the ideal mathematical model of the wireless energy transfer system can be summarized as follows:

(4) $$\eta =\frac{\omega M^{2}\frac{R_{L}}{L_{1}}}{\omega M^{2}\frac{R_{L}}{L_{1}}+\omega M^{2}\frac{r_{2}}{L_{1}}+\left[{\left(\omega L_{2}+X_{L}\right)}^{2}+{\left(r_{2}+R_{L}\right)}^{2}\right]\frac{r_{1}}{\omega L_{1}}}$$

### Modeling optimization of visual prosthesis wireless energy transfer system

**Figure 2:**
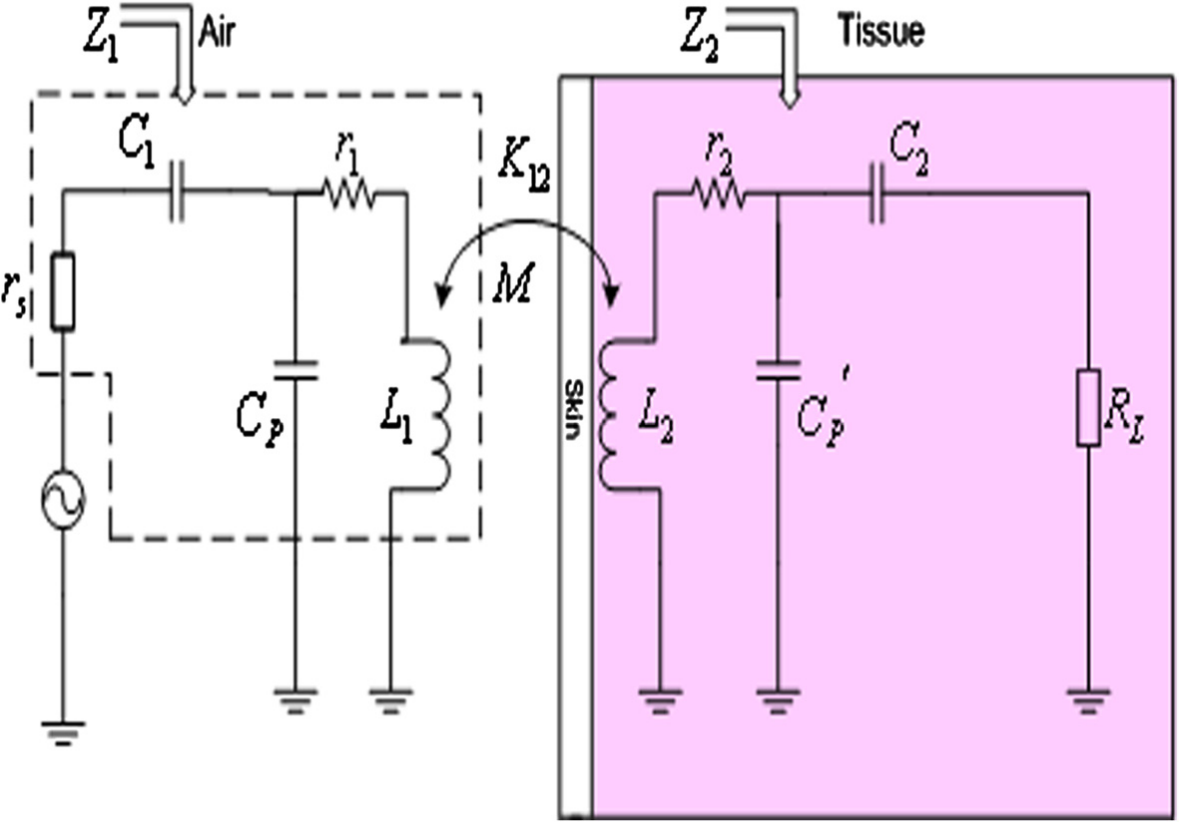
**The visual prosthesis wireless energy transmission system.** Figure 2 shows the equivalent circuit of the visual prosthesis wireless energy transfer system proposed in this paper.

During the modeling optimization in this paper, the impacts of the physical parameters in the actual circuits to the wireless energy transfer are fully considered without taking ideal assumptions, and especially the impact of biological tissue is taken account. Figure 2 shows the equivalent circuit of the perfect visual prosthesis wireless energy transfer system proposed in this paper. Compared with the ideal model shown in Figure 1, the internal resistance of power source *r*_*s*_ and the parasitic capacitance of the primary coil *C*_*p*_ are added in the primary coil circuit impedance *Z*_1_. It’s worth to note that in this paper we will use the vector network analyzer to measure the wireless energy transfer efficiency of the system, for the characteristics of the equipment, here we have *r*_*s*_ = *R*_*L*_ = 50*Ω*, and for the actual application, the load resistance *R*_*L*_ should be the equivalent load of the circuit in vivo. In the secondary coil circuit impedance *Z*_2_, the biological capacitance $C_{p}^{\prime }$ proposed in this paper is added. When the secondary coil is implanted into the body, the air medium is replaced by the biological tissue medium (the relative dielectric constant of human biological medium *ε*_*m*_ >> 1), in this paper the capacitance produced by the biological medium is defined as the biological capacitance. In addition, in order to improve the energy transfer efficiency, the resonant match capacitances *C*_1_ and *C*_2_ are added in the primary side and the secondary side respectively.

#### the planar spiral inductance coil *L*

**Figure 3:**
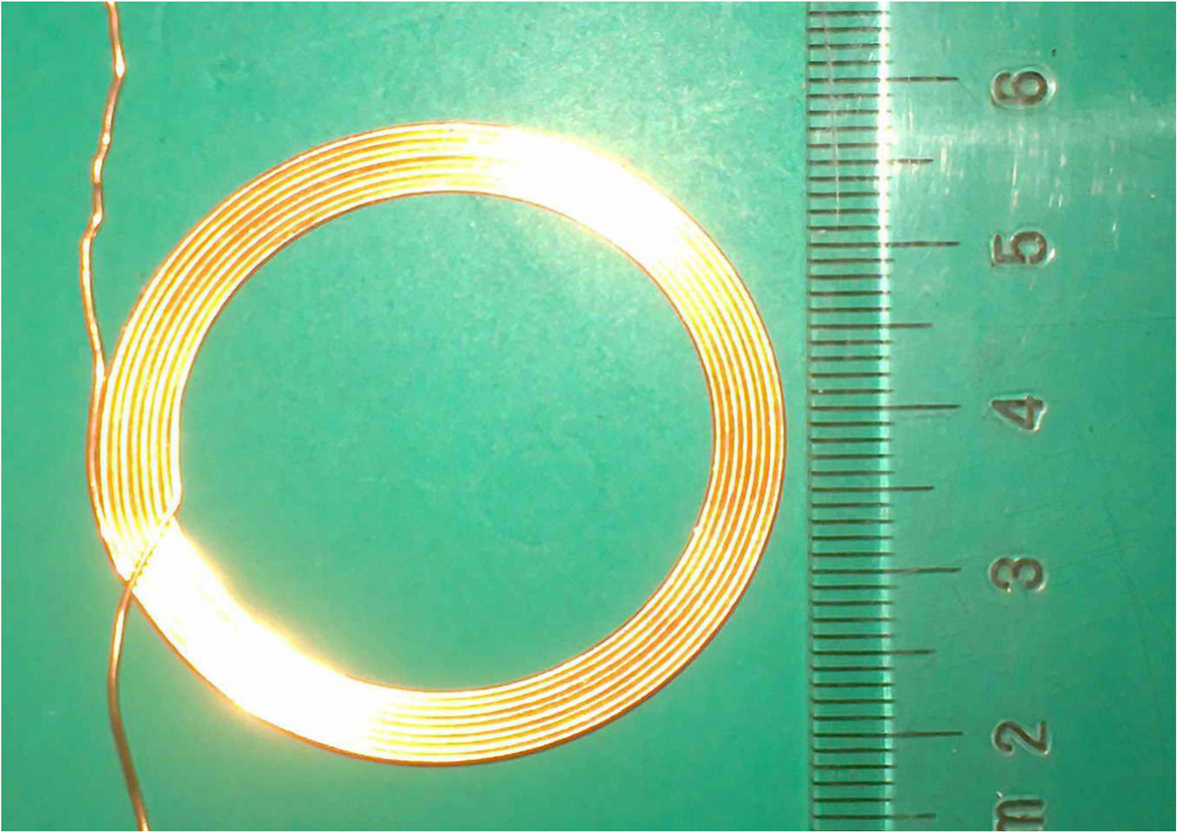
**The planar spiral inductance coil.** The coupling coils used in this system are the planar spiral inductance coils.

The coupling coils used in this system are the planar spiral inductance coils, the shape is shown in Figure 3.

Such Inductance coils are widely used in wireless charger, card reader, IC, ID card, which have the following physical properties: 

1. They can be manufactured conveniently and they are economical and durable;

2. Good flexibility, easy to be implanted.

Based on large number of experimental data statistics, the planar spiral coil inductance empirical formula [10] is adopted here:

(5) $$L=\frac{r^{2}n^{2}}{(2r+2.8d)*10^{5}}$$

From above formula (5), we can get that the parameters affecting the coil inductance can be summarized as:

Coil winding turns *n*;

Average coil winding radius *r* (the average of the outer radius and inner radius, unit: m);

Coil winding depth *d* (outer radius minus inner radius, unit: m).

In this paper, the size of the primary and secondary spiral coil (Figure 3) is the same. Number of turns *n* is 8, average winding radius *r* is 17.08mm, and winding depth *d* is 4.34mm. According to the proposed model we can obtain the planar spiral coil inductance that is *L*_1_ = *L*_2_ = 4.031*μ*H.

#### the mutual inductance *M*

When the central axis of the primary coil and the secondary coil are aligned, according to Maxwell’s equation, the mutual inductance value *M*_*ij*_ between any pair of parallel single-turn coils with the radius of *r*_*i*_, *r*_*j*_ can be expressed as equation (6) [6]:

(6) $$M_{\text{ij}}=\frac{2\mu }{\alpha }\sqrt{r_{i}.r_{j}}\;\left[\left(1-\frac{\alpha^{2}}{2}\right)K(\alpha )-E(\alpha )\right]$$

where $\alpha =2\sqrt{\frac{r_{i}.r_{j}}{{\left(r_{i}+r_{j}\right)}^{2}+D^{2}}}$;

*K*(*α*) and *E*(*α*) are class I and II of complete elliptic integral respectively;

$$\begin{array}{l} \text{Class I of elliptic integral expression is:}\;K(\alpha )=\int_{0}^{1}\frac{\text{dt}}{\sqrt{\left(1-t^{2})(1-\alpha^{2}t^{2}\right)}}\;\text{dt}\\ \text{Class II of elliptic integral expression is:}\;E(\alpha )=\int_{0}^{1}\frac{\sqrt{1-\alpha^{2}t^{2}}}{\sqrt{1-t^{2}}}\text{dt} \end{array}$$

*D* is the distance between the coils; *μ* is the transfer medium magnetic permeability between the coils.

In summary, when the turns of the primary coil are *n*_1_, the turns of the secondary coils are *n*_2_, the mutual inductance *M* between two coils can be expressed as:

(7) $$M=\overset{n_{1}}{\underset{i=1}{\sum }}\overset{n_{2}}{\underset{j=1}{\sum }}M_{\text{ij}}(r_{i},r_{j},D)$$

As the coil is shown in Figure 3, the inner diameter is 14.91mm; the diameter of each turn is 0.62mm; the vacuum magnetic permeability is *μ*_0_ = 4*π* ∗ 10^-7^*H* / *m*; the relative magnetic permeability of air and biological tissue is *μ*_*r*_ ≈ 1. Substituting into this model, when the distance between the coils *D* = 1cm, the mutual inductance *M* = 1.0209*μ*H; when the distance between the coils *D* = 2cm, the mutual inductance *M* = 0.42134*μ*H.

#### the inductance coil high-frequency equivalent series resistance *R*

Under high frequencies condition, because the inductance coil resistance is influenced by the skin effect, the proximity effect, the eddy current effect and many other effects, the impedance is much larger than the intrinsic resistance of coil at *DC*. Typically, the coil loss resistance under high frequency can break down into Ohm loss resistance *R*_*o*_ and radiation loss resistance *R*_*r*_[3,11]. In this paper, the energy transfer frequencies are in 1-50MHz, resulting in *R*_*r*_ << *R*_*o*_, so the radiation loss impedance *R*_*r*_ can be negligible.

(8) $$R_{o}=\sqrt{\frac{\omega \mu_{0}}{2\sigma }}\frac{l}{2\pi r^{\prime }}=\sqrt{\frac{\omega \mu_{0}}{2\sigma }}\frac{\text{nr}}{r^{\prime }}$$

In above formula, *μ*_0_ is the vacuum magnetic permeability; *σ* is the conductivity; *l* is the wire length; *r*^′^ is the radius of the wire; *n* is the number of the coil turns; *r* is the average radius of the coils; *ω* is the angular frequency.

In our system, the coil conductivity *σ* = 5.9 ∗ 10^7^S/m; the average radius *r* = 17.08mm; turns *n* = 8; the equivalent series resistance is changed with frequency, for example, when *ω* = 35.35534rad/s that is *f* = 5.63MHz, the coil equivalent series resistance *R*_*o*_ = 0.2705 *Ω*.

#### the inductance coil parasitic capacitance *C*_*p*_

**Figure 4:**
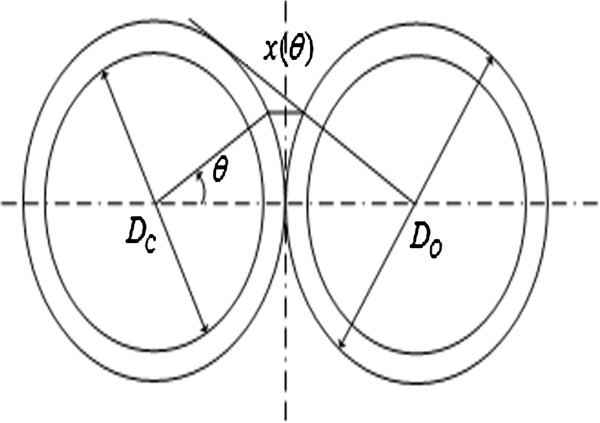
The cross-sectional view between two adjacent turns of the coil.

Figure 4 is the cross-sectional view between two adjacent turns of the coils. In the figure, *D*_*c*_ means the bare wire diameter, *D*_*o*_ means the wire diameter with the insulating layer, and *x*(*θ*) means the air gap between two adjacent wires. The relationship between *x*(*θ*) and other parameters could be given as

(9) $$x(\theta )=D_{o}(1-\cos\theta )$$

The parasitic capacitance *C*_*tt*_ between two adjacent turns of the coil can be equivalent to the insulation layer dielectric equivalent capacitance *C*_*ttc*_ in series with the inter-insulation layer air medium equivalent capacitance *C*_*ttg*_[12].

The insulation layer dielectric equivalent capacitance per unit angle can be expressed as:

(10) $$dC_{\text{ttc}}=\frac{\varepsilon_{r}\varepsilon_{0}l_{t}}{2\ln\frac{D_{o}}{D_{c}}}d\theta$$

*ε*_*r*_ is the relative dielectric constant of the insulation layer;

*l*_*t*_ is the corresponding effective length of two adjacent turns of the coil;

The inter-insulation layer air medium equivalent capacitance per unit angle can be expressed as:

(11) $$dC_{\text{ttg}}=\varepsilon_{0}\frac{l_{t}D_{o}}{2x(\theta )}=\varepsilon_{0}\frac{l_{t}}{2(1-\cos\theta )}d\theta$$

The equivalent capacitance per unit angle can be expressed as:

(12) $$dC_{\text{eq}}(\theta )=\frac{dC_{\text{ttc}}dC_{\text{ttg}}}{dC_{\text{ttc}}+dC_{\text{ttg}}}=\frac{\varepsilon_{0}l_{t}}{2}\frac{1}{1+\frac{1}{\varepsilon_{r}}\ln\frac{D_{o}}{D_{c}}-\cos\theta }d\theta$$

The equivalent capacitance between two adjacent turns of the coil can be expressed as:

(13) $$\begin{array}{lll} C_{\text{tt}} & =2\int_{0}^{\frac{\pi }{6}}\frac{\varepsilon_{0}l_{t}}{2}\frac{1}{1+\frac{1}{\varepsilon_{r}}\ln\frac{D_{o}}{D_{c}}-\cos\theta }d\theta \;\text{du}\; & \;\\ & =\varepsilon_{0}l_{t}\frac{2\varepsilon_{r}\arctan\left[\frac{\left(-1+\sqrt{3}\right)\left(2\varepsilon_{r}+\ln\frac{D_{o}}{D_{c}}\right)}{\left(1+\sqrt{3}\right)\sqrt{\ln\frac{D_{o}}{D_{c}}\left(2\varepsilon_{r}+\ln\frac{D_{o}}{D_{c}}\right)}}\right]}{\sqrt{\ln\frac{D_{o}}{D_{c}}\left(2\varepsilon_{r}+\ln\frac{D_{o}}{D_{c}}\right)}}\; \end{array}$$

By measuring the selected planar spiral coil in this paper we can get the bare wire diameter *D*_*c*_ = 0.575mm, the wire diameter including the insulation layer *D*_*o*_ = 0.620mm, the vacuum dielectric constant *ε*_0_=8.85∗10^-12^F/m, the enameled wire insulation layer relative dielectric constant *ε*_*r*_ ≈ 3.5 [13], the innermost effective corresponding length between two adjacent turns of the coil *l*_*t*_ = 97.3871mm. According to the model, the parasitic capacitance between the innermost two turns of the coil can be obtained *C*_*tt*_ = 9.9399pF; Similarly, the parasitic capacitance between each two adjacent turns of the coil can be evaluated.

The equivalent parasitic capacitance between each two adjacent turns of the multi-turn coil also can be expressed as equation (13), and the capacitances between the turns of the coil are connected in series, in other words, the lumped equivalent capacitance of the n-turn coil equals to the n-1 inter-turn capacitances in series.

(14) $$C_{p}=\frac{1}{\frac{1}{C_{12}}+\frac{1}{C_{23}}+\ldots +\frac{1}{C_{(n-1)n}}}$$

#### The Modeling of the Biological Capacitance $C_{p}^{\prime }$

The biological capacitance $C_{p}^{\prime }$ can be equivalent to the insulation layers dielectric equivalent capacitance *C*_*ttc*_ in series with the inter-insulation layer biological tissue dielectric equivalent capacitance *C*_*ttm*_(the relative dielectric constant of the biological tissues is represented by *ε*_*m*_). The inter-insulation layer biological tissue dielectric equivalent capacitance per unit angle could be expressed as:

(15) $$dC_{\text{ttm}}=\varepsilon_{0}\varepsilon_{m}\frac{l_{t}D_{o}}{2x(\theta )}=\varepsilon_{0}\varepsilon_{m}\frac{l_{t}}{2(1-\cos\theta )}d\theta$$

Connect it in series with the insulation layer dielectric equivalent capacitance per unit angle *C*_*ttc*_, then the biological capacitance per unit angle can be obtained:

(16) $$dC_{\text{eq}}^{\prime }(\theta )=\frac{dC_{\text{ttc}}dC_{\text{ttm}}}{dC_{\text{ttc}}+dC_{\text{ttm}}}=\frac{\varepsilon_{0}l_{t}}{2}\frac{1}{\frac{1}{\varepsilon_{m}}+\frac{1}{\varepsilon_{r}}\ln\frac{D_{o}}{D_{c}}-\frac{1}{\varepsilon_{m}}\cos\theta }d\theta$$

The biological capacitance between two adjacent turns of the coil is expressed as:

(17) $$C_{\text{tt}}^{\prime }=2\int_{0}^{\frac{\pi }{6}}dC_{\text{eq}}^{\prime }(\theta )=\varepsilon_{0}l_{t}\frac{2\varepsilon_{r}\varepsilon_{m}\arctan\left[\frac{\left(-1+\sqrt{3}\right)\varepsilon_{m}\ln\frac{D_{o}}{D_{c}}}{\left(1+\sqrt{3}\right)\sqrt{\left(2\varepsilon_{r}+\varepsilon_{m}\ln\frac{D_{o}}{D_{c}}\right)\varepsilon_{m}\ln\frac{D_{o}}{D_{c}}}}\right]}{\sqrt{\left(2\varepsilon_{r}+\varepsilon_{m}\ln\frac{D_{o}}{D_{c}}\right)\varepsilon_{m}\ln\frac{D_{o}}{D_{c}}}}$$

The value of relative dielectric constant of the biological tissues is different for each one and it could be decrease slowly with the increase of the frequency when the frequency arrives several MHz [13,14]. In this paper we take the relative dielectric constant *ε*_*m*_ ≈54 [14], the biological capacitance of the innermost two turns *Ctt*′=20.192pF. Similarly,

(18) $$C_{p}^{\prime }=\frac{1}{\frac{1}{C_{12}^{\prime }}+\frac{1}{C_{23}^{\prime }}+\ldots +\frac{1}{C_{(n-1)n}^{\prime }}}$$

So, the modeling built in this paper can be available not only for the general air medium wireless energy transfer system but also for the biological tissue medium wireless energy transfer system. Formula (14) is used in general air medium and formula (18) is used in biological tissue medium.

## Results

### Simulation and analysis

**Figure 5:**
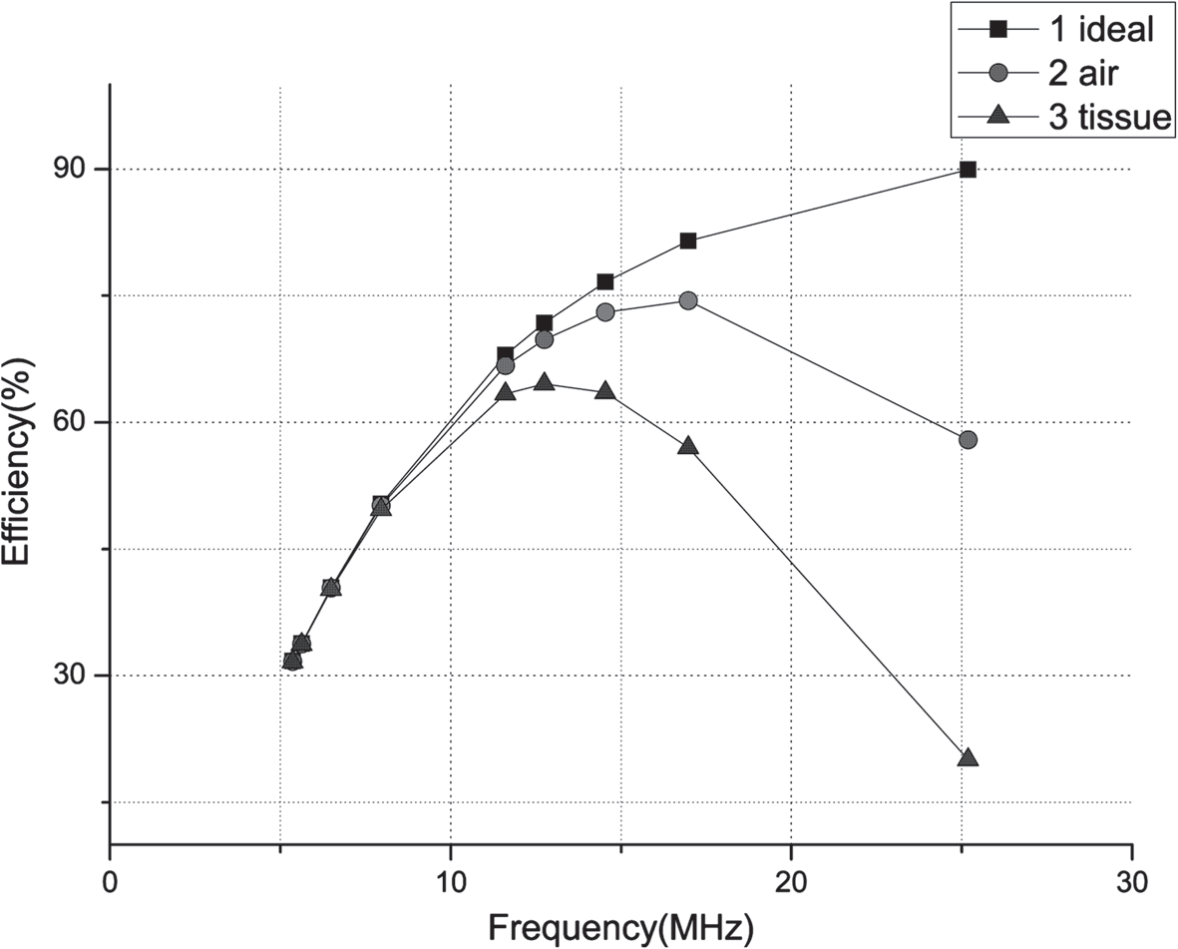
**The comparison chart of the energy transfer efficiency simulation (the distance between the coils D = 1cm).** The “Frequency - Efficiency” curves about the ideal model, the optimal model of air medium and biological medium are shown in Figure 5.

Using the optimal visual prosthesis energy transfer model proposed in this paper, we get the energy transfer efficiency at different frequency point (the distance of the primary coil and the secondary coil is 1cm), the “Frequency - Efficiency” curves about the ideal model, the optimal model of air medium and biological medium are shown in Figure 5.

The three curves in Figure 5 show energy transfer efficiency versus the frequency by using the resonance method. Among them, the first one is the curve of the ideal model, the second one is the curve of the optimal model with air medium, and the third curve is the optimal model with biological tissue medium.

From Figure 5 we can find that at the low frequencies, the three curves are almost overlapped, while with the increase of frequency, the difference between each other is increasing. The reason is that when the coil inductance is fixed, the resonance frequency in the low frequency band needs the larger coupling capacitance, the influence of the air medium inductance parasitic capacitance and the biological capacitance is negligible, so, the energy transfer efficiency of the model before and after optimization is basically equal. However, with the resonant frequency increases, the required matching capacitance gradually reduces, when it reduces to a certain extent, the influence of the inductance parasitic capacitance and its biological capacitance becomes significant, so the trend of three curves become discrete.

### Experimental verification

**Figure 6:**
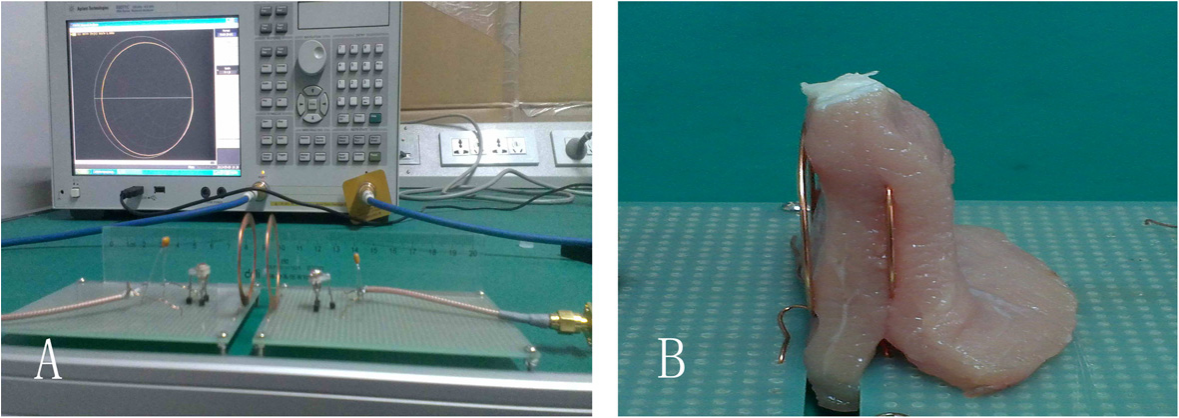
**The photos of the experimental device for measurement. A**-The experimental instrument and the vector network analyzer Agilent E5071C; **B**-The picture of the secondary coil embedded by biological tissue.

The photos of the experimental devices for measurement is shown in Figure 6, the energy transfer efficiency is measured by the vector network analyzer Agilent E5071C. The influence of the human biological tissue on the energy transfer efficiency is simulated by using the fresh lean from the intraday slaughtered pig wrapping tightly around the secondary coil.

**Figure 7:**
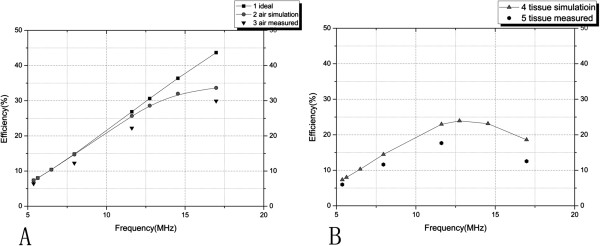
**The comparison chart of the simulation and the experimental measurement (D=2cm). A**-The comparison of measurement data in air medium with simulation data using ideal model and optimal model; **B**-The comparison of measurement data in biological medium with simulation data using optimal model simulation.

Figure 7 is the situation that the energy transfer efficiency varies with the frequency. In order to reflect the versatility of this model, in Figure 7 the distance between the primary coil and the secondary coil is changed into 2cm. Figure 7A is the comparison chart of the ideal model, the optimal model in the air medium and the measured data. It can be seen from Figure 7A that the modeling simulation results (curve 2) in the air medium is more consistent with the experimental data (the point curve 3). The ideal model (curve 1) which ignores the parasitic capacitance is basically consistent with the measured data at the low frequencies, but the extent of error in the high frequency band cannot be tolerated, therefore the optimization modeling theory of this paper is closer to the actual situation. Figure 7B is the comparison chart of the simulation data in the biological media and the measured data, we can see that the trend of the two curves basically coincide, the extent of error range is within the reasonable range, which verifies the correctness of the model.

**Figure 8:**
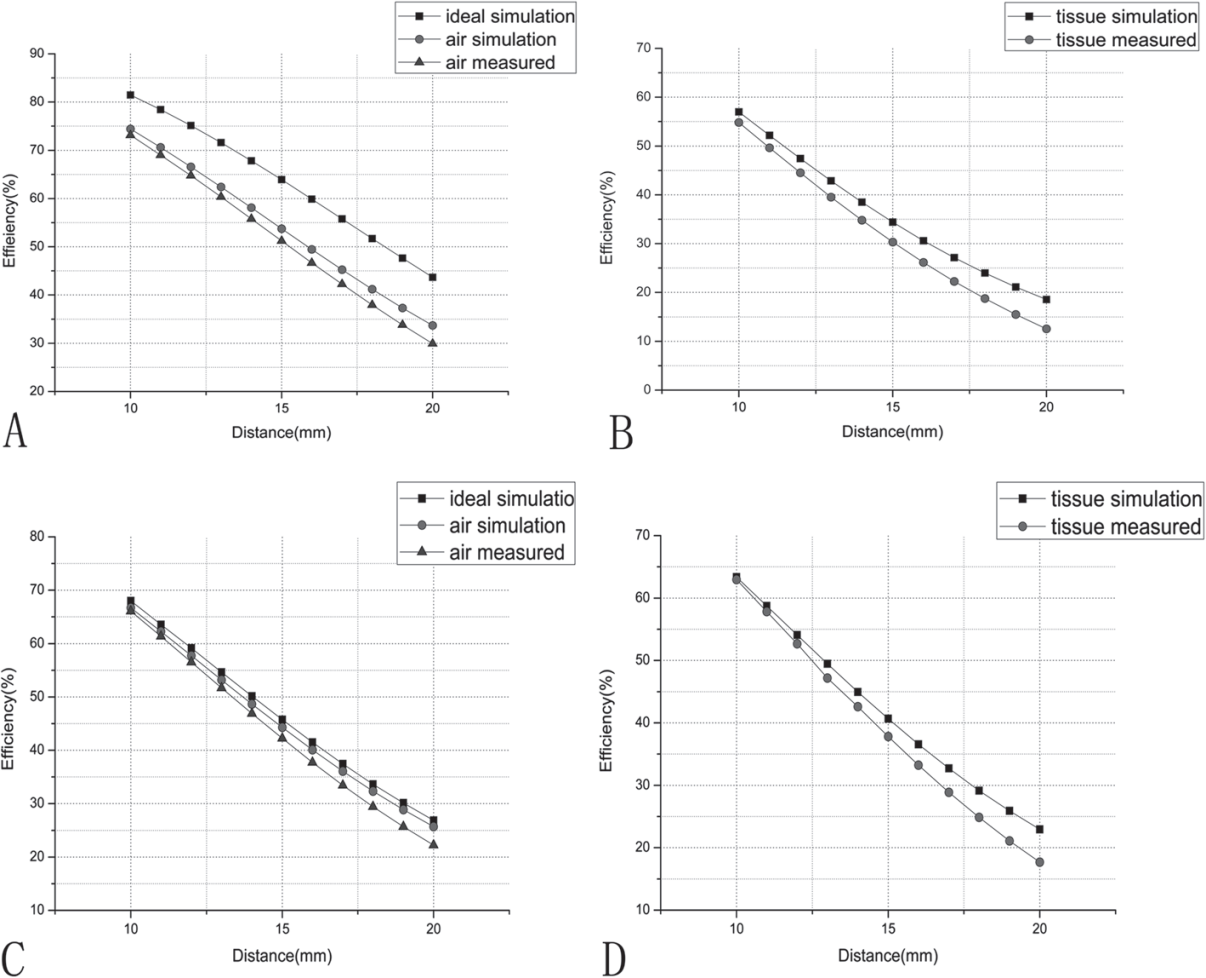
**The diagram of the energy transmission efficiency vs. the distance between the coils. A**-The curves in the air medium at 16.97MHz; **B**-The curves in the biological medium at 16.97MHz; **C**-The curves in the air medium at 11.61MHz; **D**-The curves in the biological medium at 11.61MHz.

The inductance values of the planar spiral inductance coils used in this paper are *L*_1_ = *L*_2_ = 4.031*μ*H, when the match capacitances are 22pF and 47pF, the resonant frequencies are 16.97MHz and 11.61MHz respectively. Figure 8 shows that the energy transfer efficiency in the air medium and in the biological tissue medium varies with the transfer distances at two different frequencies 16.97MHz and 11.61MHz. From Figure 8 we can find that the air medium modeling highly coincides with the actual measurement data, the biological tissue medium modeling also basically coincides with the measured data within the distance of 1-2cm. According to clinical medicine experience, the distance between the secondary visual prosthesis reception coil which implant into the patient’s body and the primary coil outside the body is usually 1-2cm. Therefore, this model meets the requirements of the implantation depth range for the actual clinical application.

## Conclusions

Based on the physical characteristics of the biological tissue, optimization modeling of the visual prosthesis wireless energy transfer system is established in this paper. During the optimization modeling, the influence of the parasitic capacitance of the coil and the biological capacitance to the energy transfer efficiency is considered fully, which greatly improves the matching degree of the theoretical modeling and the measured data. The optimal modeling has a higher guiding significance for the actual system design.

## Competing interests

The authors declare that they have no competing interests.

## Authors’ contributions

LXP, YY and GY have equally contributed to the manuscript; both were also involved in the design of the modeling study and data analysis. All authors read and approved the final manuscript.

## Authors’ information

LXP is a lecturer and doctoral candidate in Xi’an University of Technology. His research subjects are Visual prosthesis wireless energy and data transfer. YY is a professor in Xi’an University of Technology. And she is also the vice dean of graduate school of the university. She is supported by National Natural Science Foundation of China. GY is a professor and academic leader of electronics science and technology subject in Xi’an University of Technology.
